# Factors influencing the completion of advance directives in cancer patients: a descriptive survey

**DOI:** 10.1186/s12904-025-01955-4

**Published:** 2025-11-28

**Authors:** Wonjeong Hwang, Jiyoung Do

**Affiliations:** 1 Department of Nursing, Pusan National University Hospital, 179 Gudeok-ro, Seo-gu, Busan, 49241 South Korea; 2 College of Nursing, Catholic University of Pusan, 74 Oryndae-ro, Geumjeong-gu, Busan, 46252 South Korea

**Keywords:** Advance directives, Neoplasms, Attitude to death, Patient autonomy

## Abstract

**Aim:**

This study aimed to investigate the factors associated with the completion of advance directives among cancer patients.

**Background:**

Despite legislation to support end-of-life decision-making, the completion rate of advance directives (AD) in South Korea remains low. In many cases, family members make end-of-life decisions on behalf of patients. To promote patient autonomy and reduce family burden, early initiation of AD discussions is essential.

**Methods:**

Survey data on demographics and factors related to AD completion were collected through a written survey administered by the researchers from 148 cancer patients at a tertiary hospital between November 2, 2023, and March 10, 2024. Data analysis included frequency, percentage, mean, standard deviation, χ²-test, independent t-test, Pearson’s correlation coefficient, and multivariate logistic regression.

**Results:**

Compared to patients aged 70 and older, those under 60 were 0.18 times as likely, and those aged 60 to 69 were 0.27 times as likely, to complete an AD. Patients with a middle school education or less were 12.46 times more likely to complete an AD than those with higher education levels. Having prior experience discussing the withdrawal of life-sustaining treatment during the death of a loved one increased the likelihood of completing an AD by 18.64 times. Additionally, each 1-point increase in psychological and formal Readiness for Death was associated with a 6.78-fold increase in the likelihood of completing an AD. Age, education level, prior experience, and particularly Readiness for Death were found to be associated with AD completion among cancer patients.

**Conclusions:**

AD completion among cancer patients was associated with age, education level, prior discussion of LST withdrawal, and Readiness for Death. These findings highlight factors that may inform future interventions to support patient autonomy via increased uptake of ADs.

## Introduction

### Definition and purpose of advance directives

Advance Directives (ADs) are documents that allowing individuals to express their preferences regarding life-sustaining treatments and hospice care in preparation for end-of-life decisions [1]. These documents recognize the patient’s right to self-determination in decisions about life-sustaining treatments, thereby facilitating a natural death [1, 2].

### Legal and cultural influences on advance directive completion

In South Korea, the enactment of the Life-Sustaining Treatment Decisions Act in 2018 increased public awareness of ADs, but the overall completion rate remains low at 6% as of 2023 [1]. In Korean culture, people tend to avoid open discussions about death [3], and critical medical decisions regarding end-of-life care are often made within a family-centered framework [4]. These cultural factors significantly affect patients’ willingness and ability to complete ADs. Taiwan, which shares similar cultural attitudes toward death and family-centered decision-making, legalized ADs in 2019, allowing individuals to refuse life-sustaining treatment not only at the end of life but also in conditions such as irreversible coma or advanced dementia [5]. Nevertheless, the completion rates for AD remain low [6]. In contrast, the United States, where individual autonomy is emphasized over family consensus, reports much higher completion rates of about 30–40% [7]. These cross-national differences suggest that both legal frameworks and cultural factors play critical roles in shaping AD completion. Research within Korea’s unique legal and cultural context to identify effective strategies that can support patient autonomy and improve AD completion rates is needed.

### Importance of early preparation for death

The primary aim of an AD is to empower patients to experience a dignified death [8]. By safeguarding the right to self-determination regarding life-sustaining treatments, ADs contribute to improving the quality of life for patients [9]. Therefore, supporting individuals and their families in preparing end-of-life plans during healthier stages is essential [9]. Patients with cancer face extended illness trajectories and more likely to contemplate death during treatment [10]. Preparing for death proactively has been shown to enhance life satisfaction [11]. For patients with cancer, addressing end-of-life anxiety through active preparation is critical in alleviating both emotional and physical distress, enabling a more meaningful final phase of life [12].

### Readiness for death

Readiness for Death encompasses psychological and formal readiness, aiming to accept death while preserving dignity at the end of life [11]. Psychological Readiness for Death includes adopting an accepting attitude toward death through activities such as completing an AD or participating in death education programs. Completing an AD while still healthy not only reduces death anxiety but also promotes a sense of tranquility, enabling individuals to prepare meaningfully for life’s final stage [13].

### Self-Esteem and its influence on AD completion

Cancer diagnosis and treatment can negatively impact patients’ physical and psychological well-being, leading to emotional distress and altered self-esteem [14]. Self-esteem, defined as an individual’s evaluation of their self-worth, influences how they perceive and cope with illness and treatment challenges [15]. Higher self-esteem in patients with cancer correlates with reduced fear of cancer recurrence and realistic disease perceptions, which facilitate active coping strategies [16]. Self-esteem also positively affects attitudes toward discontinuing life-sustaining treatments, suggesting that enhancing self-esteem may influence patients’ decision-making processes regarding their own end-of-life care [11].

### Research gaps and study aim

Previous studies have identified good death perceptions [3], attitudes toward a dignified death [17], and negative attitudes toward death [13] as being relevant to AD completion. However, most studies have focused on groups such as community-dwelling older adults [13] or general adults [17], with limited research on patients themselves. Moreover, few studies have compared patients who have completed ADs with those who have not. Considering the rising number of cancer survivors [18], their unique psychosocial challenges [10, 12], and the low AD completion rates in South Korea despite a legal framework, research in this population is warranted.

In this study, we aimed to explore differences in Readiness for Death, attitudes toward a dignified death, and self-esteem between patients with cancer who have completed AD and those who have not. By identifying the factors associated with AD completion, the findings of this study will inform strategies to promote AD adoption among patients with cancer, ultimately contributing to enhanced end-of-life planning.

## Methods

### Study design

This study employed a descriptive survey design to investigate the factors associated with AD completion among patients with cancer.

### Sample and data collection

The participants were patients with cancer either hospitalized or attending outpatient care at a tertiary hospital located in metropolitan city in South Korea. Participants were eligible if they were adults diagnosed with cancer, able to read and/or speak Korean, and capable of providing informed consent. Patients with terminal illness who had completed a life-extending treatment plan under the Life-Extending Treatment Decisions Act were excluded. Participants were classified into the AD completion group (AD group) or non-completion group (non-AD group) based on whether they had completed an AD.

Sample size was calculated for logistic regression, a significance level (α) of 0.05, power (1-β) of 0.80, and an odds ratio of 2.0 [19] using G*Power 3.1.9.7. 113 participants were required based on these assumptions. Previous Korean studies have reported that some older patients with cancer refused to participate in surveys addressing end-of-life issues [20]. However, in calculating the sample size of this study, we focused on potential attrition during the study, which is distinct from initial refusal. Although dropout rates of up to 33% have been reported in previous international research [21], this represents an extreme value; therefore, we conservatively applied a 25% dropout rate, taking into account the feasibility of participant recruitment at our institution. Therefore, a total of 151 participants were recruited. Ultimately, 148 questionnaires were analyzed, excluding 3 incomplete responses (dropouts). Recruitment for the non-AD group was finalized first, followed by matched recruitment for the AD group.

Data were collected from November 2, 2023, to March 10, 2024. Recruitment posters were placed in outpatient and ward areas with prior permission from hospital nursing staff and clinicians. Each questionnaire was administered face-to-face and took approximately 15 min to complete; for participants who had difficulty reading or writing, the researcher read the questions aloud. Completed questionnaires were collected in sealable envelopes to ensure that only the research team could access the contents, thereby protecting participants’ confidentiality.

### Variables and categorization

Age and education categories were set based on prior research and the mean age of the study population, which was 65.2 years. Due to limited prior research among Korean patients with cancer, we referred to domestic studies on older adults with a similar age range [22]. Age was classified as < 60 years, 60 s, and ≥ 70 years, with participants aged ≥ 80 years combined with the 70 s group due to small numbers. Education was initially collected as elementary, middle, high school, or college and above. Given the small number of participants with only an elementary education and the high proportion of older adults with middle school education or less, and referencing the 2023 National Survey on Older Adults [23] and previous research [24], education was dichotomized as middle school or less versus high school or more for analysis.

### Instruments

#### General characteristics

General characteristics were assessed using a 13-item questionnaire that included sex, age, education, religion, marital status, monthly income, experience discussing the withdrawal of life-sustaining treatment for family or acquaintances at end of life (Discussion of LST withdrawal), AD completion status, diagnosis, disease duration, current cancer stage, recurrence and metastasis history, and subjective health status.

#### Readiness for death

Readiness for Death was measured using a 10-item tool developed by Kim [11] and revised by Moon and Nam [25]. The instrument comprises two subdomains: Psychological Readiness for Death (3 items) and Formal Readiness for Death (7 items). Responses were rated on a 4-point Likert scale, with higher scores indicating greater preparedness. Cronbach’s α was 0.79 for the revised scale [25] and 0.76 for this study.

#### Attitudes toward a dignified death

Attitudes toward a dignified death were assessed using a 30-item tool developed by Cho [26] with prior approval. This scale includes five subdomains: Maintaining emotional comfort (10 items), arranging social relationship (9 items), avoiding suffering (3 items), maintaining autonomical decision making (4 items), and role preservation (4 items). Responses were rated on a 4-point Likert scale, and higher scores indicate stronger preferences for a dignified death. Cronbach’s α was 0.92 for Cho’s study [26] and 0.89 for this study.

#### Self-Esteem

Self-esteem was measured using Rosenberg’s Self-Esteem Scale [15], translated into Korean by Jeon [27]. This 10-item scale uses a 4-point Likert scale, with total scores ranging from 10 to 40, where higher scores indicate stronger preferences for self-esteem. Cronbach’s α was 0.92 for Rosenberg’s study [15], 0.89 for Jeon’s study [27], and 0.71 for this study.

### Data analysis

Data were analyzed using SPSS 27.0. Differences in general characteristics between the AD and non-AD groups were analyzed using the chi-square test. Differences in Readiness for Death, attitudes toward a dignified death, and self-esteem between groups were analyzed using frequency, mean, standard deviation, and independent t-tests. Correlations among Readiness for Death, attitudes toward a dignified death, and self-esteem were analyzed using Pearson’s correlation coefficients. Factors associated with AD completion were analyzed using multivariate logistic regression analysis.

### Ethical considerations

Ethics approval for this study was obtained from the Institutional Review Board of the authors’ affiliated institution (Approval No. CUPIRB-2023-040) Prior to the survey, the researcher provided participants with sufficient information about the purpose of the study and the data collection methods. Only those who voluntarily agreed to participate were included. Informed consent was obtained from all participants through a consent form.

## Results

### Differences in general characteristics between groups

Comparison of participant characteristics revealed significant differences between the AD group (*n* = 74) and the non-AD group (*n* = 74) in terms of age, education level, discussion of LST withdrawal, and cancer recurrence or metastasis. The AD group was predominantly aged 70 years or older (66.2%), while the non-AD group had the highest proportion of participants under 60 years of age (43.2%). The AD group’s average age was 70.86 ± 7.44 compared to the non-AD group’s average age of 59.6 ± 11.88. Regarding education level, 52.7% of the AD group had middle school education or below, whereas 83.8% of the non-AD group had high school education or above. Discussion of LST withdrawal was more common in the AD group (83.8%) than in the non-AD group (27.0%) (χ²=48.24, *p* <.001). And the AD group also had a higher rate of cancer recurrence or metastasis (67.6%) than the non-AD group (50.0%) (χ²=4.71, *p* =.030) (Table 1).

**Table 1:** Participant characteristics (*N *= 148)

	Categories	*n*(%) or M ± SD		AD Group (*n* = 74)	Non-AD Group (*n* = 74)	χ²	*p*
Sex	M	87(58.8)		44(59.5)	43(58.1)	0.03	0.867
	F	61(41.2)		30(40.5)	31(41.9)		
Age(yr)	< 60	38(25.7)		6(8.1)	32(43.2)	38.96	< 0.001
	60 ~ < 70	47(31.8)		19(25.7)	28(37.8)		
	≥ 70	63(42.6)		49(66.2)	14(18.9)		
		65.26 ± 11.37		70.86 ± 7.44	59.6 ± 11.88		
Education	≤Middle school		51(34.5)	39(52.7)	12(16.2)	21.81	< 0.001
	≥High school	97(65.5)		35(47.3)	62(83.8)		
Religion	Yes	76(51.4)		43(58.1)	33(44.6)	2.70	0.100
	No	72(48.6)		31(41.9)	41(55.4)		
Spouse	Yes	102(68.9)		53(71.6)	49(66.2)	0.51	0.477
	No	46(31.1)		21(28.4)	25(33.8)		
Monthly income	< 100	71(48.0)		39(52.7)	32(43.2)	1.33	0.249
	≥ 100	77(52.0)		35(47.3)	42(56.8)		
Discussion of LST withdrawal	Yes	82(55.4)		62(83.8)	20(27.0)	48.24	< 0.001
	No	66(44.6)		12(16.2)	54(73.0)		
Diagnosis	Gastroenteric	47(31.8)		28(37.8)	19(25.7)	6.39	0.094
	Lung	43(29.1)		20(27.0)	23(31.1)		
	Hematologic	37(25.0)		13(17.6)	24(32.4)		
	Other	21(14.2)		13(17.6)	8(10.8)		
Duration after	< 12	58(39.2)		29(39.2)	29(39.2)	5.81	0.121
diagnosis	12 ~ 35	51(34.5)		20(27.0)	31(41.9)		
(month)	36 ~ 59	17(11.5)		10(13.5)	7(9.5)		
	≥ 60	22(14.9)		15(20.3)	7(9.5)		
		30.73 ± 45.42		36.53 ± 49.21	24.93 ± 40.81		
Recurrence /Metastasis	Yes	87(58.8)		50(67.6)	37(50.0)	4.71	0.030
	No	61(41.2)		24(32.4)	37(50.0)		
Subjective	Good	20(13.5)		8(10.8)	12(16.2)	0.93	0.336
health status	No	128(86.5)		66(89.2)	62(83.8)		

Discussion of LST withdrawal = Experience discussing the withdrawal of life-sustaining treatment for family or acquaintances at end of life

### Differences in variables between groups

Table 2 presents the differences in Readiness for Death, attitudes toward a dignified death, and self-esteem between the two groups.

**Table 2:** Differences of variables between groups (*N* = 148)

Variable	Total M ± SD	AD Group M ± SD	Non-AD group M ± SD	t	*p*
Readiness for Death	2.79 ± 0.45	3.02 ± 0.40	2.55 ± 0.37	7.35	< 0.001
Psychological Readiness for Death	3.14 ± 0.49	3.32 ± 0.47	2.96 ± 0.44	4.86	< 0.001
Formal Readiness for Death	2.64 ± 0.54	2.89 ± 0.50	2.38 ± 0.45	6.50	< 0.001
Attitudes Toward a Dignified Death	3.12 ± 0.32	3.26 ± 0.29	2.97 ± 0.28	5.99	< 0.001
Maintaining autonomical decision making	3.38 ± 0.48	3.53 ± 0.47	3.23 ± 0.46	3.96	< 0.001
Arranging social relationship	3.18 ± 0.36	3.33 ± 0.34	3.03 ± 0.32	5.53	< 0.001
Maintaining emotional comfort	3.04 ± 0.36	3.16 ± 0.34	2.93 ± 0.35	3.95	< 0.001
Role preservation	3.03 ± 0.43	3.16 ± 0.39	2.91 ± 0.43	3.58	< 0.001
Avoiding suffering	2.91 ± 0.53	3.14 ± 0.43	2.68 ± 0.52	5.75	< 0.001
Self Esteem	2.84 ± 0.44	2.80 ± 0.46	2.88 ± 0.43	−1.10	0.273

Readiness for Death: On a 4-point scale, the mean score was 2.79 ± 0.45, with subdomain scores of 3.14 ± 0.49 for Psychological Readiness for Death and 2.64 ± 0.54 for Formal Readiness for Death. The AD group had significantly higher scores (3.02 ± 0.40) compared to the non-AD group (2.55 ± 0.37) (t = 7.35, *p* <.001). Subdomain scores showed significant differences, with the AD group scoring higher for both Psychological Readiness for Death (3.32 ± 0.47 vs. 2.96 ± 0.44, t = 4.86, *p* <.001) and Formal Readiness for Death (2.89 ± 0.50 vs. 2.38 ± 0.45, t = 6.50, *p* <.001).

Attitudes Toward a Dignified Death: The mean score was 3.12 ± 0.32, with subdomains ranked as follows: maintaining autonomical decision making (3.38 ± 0.48), arranging social relationship (3.18 ± 0.36), maintaining emotional comfort (3.04 ± 0.36), role preservation (3.03 ± 0.43), and avoiding suffering (2.91 ± 0.53). The AD group had significantly higher scores (3.26 ± 0.29) compared to the non-AD group (2.97 ± 0.28) (t = 5.99, *p* <.001). All subdomains showed significant differences, with the AD group scoring higher in maintaining emotional comfort (3.16 ± 0.34 vs. 2.93 ± 0.35, t = 3.95, *p* <.001), arranging social relationship (3.33 ± 0.34 vs. 3.03 ± 0.32, t = 5.53, *p* <.001), avoiding suffering (3.14 ± 0.43 vs. 2.68 ± 0.52, t = 5.75, *p* <.001), maintaining autonomical decision making (3.53 ± 0.47 vs. 3.23 ± 0.46, t = 3.96, *p* <.001), and role preservation (3.16 ± 0.39 vs. 2.91 ± 0.43, t = 3.58, *p* <.001).

### Factors associated with AD completion

To identify factors associated with AD completion, multivariate logistic regression was conducted, including variables that showed significant differences between the groups (age, education level, discussion of LST withdrawal, recurrence/metastasis, Readiness for Death, attitudes toward a dignified death, and self-esteem). AD completion was set as the dependent variable (1 = completion, 0 = non-completion). The model’s goodness-of-fit was verified with the Hosmer-Lemeshow test (χ²=12.28, *p* =.139), and its explanatory power was 72.4% based on Nagelkerke R².

Participants under 60 years were 0.18 times as likely (95% CI = 0.04–0.90, *p* =.037), and those in their 60 s were 0.27 times as likely (95% CI = 0.08–0.91, *p* =.035) to complete AD compared to those aged 70 years or older. Participants with middle school education or lower were 12.46 times more likely to complete AD than those with high school education or higher (95% CI = 2.86–54.35, *p* =.001). Participants with discussion of LST withdrawal were 18.64 times more likely to complete an AD compared to those without such discussion (95% CI = 4.96–70.02, *p* <.001). A 1-point increase in Readiness for Death increased the likelihood of AD completion by 6.78 times (95% CI = 1.25–36.83, *p* =.027) (Table 3).

**Table 3:** Factors associated with AD completion (*N* = 148)

Characteristics/Variables	Categories	B	*p*	OR	95% CI
Age (ref.: ≥70)	< 60	−1.73	0.037	0.18	0.04 ~ 0.90
	60 ~ < 70	−1.31	0.035	0.27	0.08 ~ 0.91
Education (ref.: ≥High school)	≤Middle school	2.52	0.001	12.46	2.86 ~ 54.35
Discussion of LST withdrawal (ref.: No)	Yes	2.92	< 0.001	18.64	4.96 ~ 70.02
Recurrence/Metastasis (ref. : No)	Yes	0.40	0.464	1.49	0.51 ~ 4.36
Readiness for Death		1.91	0.027	6.78	1.25 ~ 36.83
Attitudes Toward a Dignified Death		2.06	0.096	7.89	0.70 ~ 89.71
Self Esteem		−0.80	0.273	0.44	0.10 ~ 1.89

Hosmer & Lemeshow test : χ²=12.28, *p* =.139

Nagelkerke’s R²=0.724

*AD* Advance Directives, *OR * Odds ratio, *CI * Confidence Interval, *ref.* Reference, Discussion of LST withdrawal, Experience discussing the withdrawal of life-sustaining treatment for family or acquaintances at end of life

## Discussion

This study was conducted to identify factors associated with AD completion among patients with cancer. Logistic regression analysis was used to identify factors affecting AD completion, and age (*p* =.037), education level (*p* =.001), discussion of LST withdrawal (*p* <.001), and Readiness for Death (*p* =.027) were found to be significantly associated with AD completion.

The average Readiness for Death among participants was 2.79 out of 4, higher in the AD group than in the non-AD group. According to a previous study [24], older adults who had thought about death were 3.53 times more likely to express an intention to complete an AD than those who had not. Similarly, older adults with greater awareness of death were more likely to consider advance care planning [28], supporting the findings of this study. Although direct comparison is difficult, patients with cancer also appear to reflect characteristics similar to older adults, repeatedly confronting death throughout diagnosis and treatment. When examining the subdomains of Readiness for Death, both groups scored higher in psychological Readiness for Death compared to formal Readiness for Death. This contrasts with previous findings [29], which reported that Korean older adults actively engage in traditional formal Readiness for Death, such as securing burial sites, preparing shrouds, and joining funeral associations. These findings may be related to the characteristics of patients with cancer, who often exhibit higher psychological Readiness for Death due to increased illness awareness from physical decline and heightened death anxiety caused by sudden diagnosis and treatment [12, 30]. Strengthening psychological Readiness for Death may help alleviate end-of-life anxiety and promote emotional acceptance of death [11]. Psychosocial programs that support spiritual readiness and self-reflection can aid these patients in preparing for death.

The average attitude toward a dignified death was 3.12 out of 4, with significantly higher scores in the AD group compared to the non-AD group. Among the subdomains, both groups rated autonomous decision-making as the most important, with the AD group scoring significantly higher than the other group. Similar findings were reported in studies on patients with cancer [31] and hospice patients [32], highlighting the importance of self-determination in death-related decisions. A patient’s right to self-determination is realized through the principle of informed consent, which serves as a critical standard in determining the continuation or withdrawal of life-sustaining treatment under the Life-Sustaining Treatment Decisions Act [2]. Thus, completing an AD while patients are cognitively capable could be a practical approach to reflecting their treatment preferences. Among other subdomains, the order of scores was social relationship regulation, followed by pain avoidance. This finding aligns with research on hospice patients [32], suggesting that prioritizing social relationship management over freedom from pain reflects the family-centered decision-making culture in South Korea. Family presence during the end-of-life stage is an essential aspect of a dignified death in Korean society [26]. Providing early information about the life-sustaining treatment system and AD can facilitate discussions between patients and families, ensuring that patient preferences are respected.

The average self-esteem score was 2.84 out of 4, with 2.80 in the AD group and 2.88 in the non-AD group, showing no significant difference. This finding contrasts with previous research, which reported a positive association between higher self-esteem and acceptance of withholding or withdrawing life-sustaining treatment [11]. However, caution is warranted when interpretating these findings, as the Cronbach’s α of the self-esteem measure in this study was relatively low at 0.71. As self-esteem reflects attitudes toward life, which may be associated with death preparedness and AD completion, further studies are recommended to explore these relationships in greater detail.

This study discussed the findings on factors associated with AD completion among patients with cancer as follows. First, patients under 60 and those in their 60 s were 0.18 and 0.27 times as likely, respectively, to complete AD compared to those in their 70s. This finding aligns with prior research indicating that older individuals have more positive attitudes toward ADs and the withdrawal of life-sustaining treatment [33]. International studies also support this result, showing that the older the patient, the more likely they are to have an AD [19, 34]. According to the National Agency for the Management of Life-Sustaining Treatment statistics from 2018 to 2023, 86.9% of individuals who registered an AD were aged 60 or older, with a particularly high registration rate among those aged 70 or above [1]. However, this study recruited patients from a single tertiary hospital, and the participation rate of younger age groups may have been relatively low; therefore, caution is needed when generalizing AD completion rates by age. Older cancer patients generally talk about death more than younger patients and desire a dignified death, but due to uncertainty about how they will die, they are willing to complete an AD [34]. While death is a universal experience, ADs are crucial tools for exercising self-determination in decisions about the withdrawal of life-sustaining treatment [33]. Therefore, efforts should be made to promote AD adoption across all age groups, ensuring that individuals have the opportunity to make informed decisions regardless of their age.

Second, patients with middle school or lower education levels were 12.46 times more likely to complete AD compared to those with high school education or higher. This finding contrasts with prior research indicating that higher education levels have significantly higher rates of completed ADs [19, 28]. This difference can be attributed to the characteristics of our study sample, with a mean age of 65.2 years and 63% of patients aged 70 or older, the tendency in Korea for older age groups to have lower education levels, and the particularly high proportion of older adults in Busan compared to the national average. However, since this study was conducted in a single tertiary hospital, and the participation rate of younger and higher-educated individuals may have been relatively low, caution is required when generalizing these results.

Third, In the AD group, 83.8% discussed the withdrawal of life-sustaining treatment with family or acquaintances at the end of life, while only 27.0% of the non-AD group did so. Moreover, patients who had discussed the withdrawal of life-sustaining treatment were 18.64 times more likely to complete AD. This finding is consistent with prior research showing that discussing life-sustaining treatment with family or acquaintances positively influences attitudes toward ADs [3, 33]. Additionally, the presence of AD completers among acquaintances has been shown to increase patients’ intent to complete AD themselves [31]. Discussions about life-sustaining treatment provide opportunities to contemplate one’s own death [33]. However, Korea has a family-centered decision-making culture [26]. Previous domestic research [31] found that 85.0% of patients with cancer had not discussed AD with their families, indicating ongoing reluctance to discuss death. Similarly, in Hong Kong, terminally ill patients tend to entrust end-of-life decisions to their families and physicians rather than expressing their own preferences [35]. Cultural factors often lead families to be reluctant to disclose diagnoses and prognoses to patients, which hinders advance care planning [35]. Therefore, patients should be encouraged to discuss life-sustaining treatment with family or acquaintances, organize their thoughts, and ensure their wishes are accurately reflected. Effective communication can facilitate the completion of an AD, reducing family guilt and ensuring that patients receive care aligned with their preferences.

Fourth, for every 1-point increase in Readiness for Death, the likelihood of completing an AD increased by 6.78 times. While few studies have directly examined the relationship between Readiness for Death and AD completion, a previous study has shown that negative attitudes toward death significantly affect the intention to complete AD [13]. Patients with cancer may initially struggle to actively discuss or prepare for death due to shock and anxiety following diagnosis. Over time, however, they become more receptive to contemplating death and addressing emotional needs [10]. Thus, approaches to fostering Readiness for Death should differ from those for the general population. For patients with cancer, Readiness for Death should emphasize forming positive attitudes toward death and integrating the experience into a meaningful continuum of life.

Completing an AD may represent an important first step in preparing for end-of-life care. In the United States, healthcare institutions routinely verify whether patients have an AD and offer guidance when one is not in place. However, in South Korea, despite the implementation of the Life-Sustaining Treatment Decision Act in 2018, no legal obligation exists to provide information about ADs, and national-level policies remain underdeveloped. Consequently, public awareness and understanding of AD are generally low. Previous research has shown that individuals who perceive greater benefits of AD completion are more likely to complete one [36]. Therefore, developing structured educational programs, along with supportive policies at both hospital and community levels, is important. Age-specific education can encourage early advance care planning, and clinical integration of treatment planning and AD counseling is necessary.

## Limitations

This study has several limitations.

First, it was conducted among patients with cancer who visited or were admitted to a single tertiary hospital. During recruitment, many potential participants either felt negative or depressed after hearing about the study topic, or had already completed an advance directive (AD) but did not consent to participate due to health-related issues. These factors may introduce potential selection bias, highlighting the need for future studies to employ random or stratified sampling methods to include a broader sample of patients with cancer.

Second, cultural factors in Korea, such as family-centered decision-making, may influence AD completion, warranting caution in interpreting the results. Similarly, in Taiwan, despite a comparable legal framework, cultural factors have been reported to result in low AD completion rates, suggesting that culture may also play a significant role in South Korea.

Third, as a descriptive survey study, causality between the identified factors and AD completion cannot be established, and the incidence or prevalence of AD completion cannot be determined. Although reporting AD completion by timing or cancer stage could be informative, this study did not address such differences. Furthermore, the reliability of the self-esteem measure was relatively low (Cronbach’s α = 0.71), necessitating caution in interpreting the results.

Therefore, future research should aim to clarify the causal relationships and the strength of associations among factors related to AD completion.

## Conclusion

This study aimed to identify the differences in demographics, Readiness for Death, attitudes toward a dignified death, and self-esteem between patients with cancer who completed an AD and those who did not, highlighting factors associated with AD completion. The results identified age, education level, discussion of LST withdrawal, and Readiness for Death as significant factors. Among these, the most associated factor was discussion of LST withdrawal. To promote AD completion, fostering a supportive social environment and institutional measures is essential. Such discussions not only uphold patient autonomy but also alleviate the burden on families. Actively preparing for death enables patients with cancer to lead a more autonomous and meaningful life. Ultimately, an AD serves as both a strategy to enhance their quality of life and a crucial starting point for end-of-life preparation. The ultimate objective of examining factors associated with AD is to enhance their actual completion rates. This study compared individuals with cancer who completed AD with those who did not, identifying key factors associated with AD completion. These findings provide practical guidance for developing tailored strategies to promote psychological Readiness for Death among patients with cancer and support open communication with their families.

## Data Availability

The datasets generated and/or analyzed during the current study are available from the corresponding author on reasonable request.
